# Incidence and Risk Factors for Varicella‐Zoster Virus‐Associated Central Nervous System Infections: A Nationwide Swedish Retrospective Case‐Control Study

**DOI:** 10.1002/jmv.70166

**Published:** 2025-01-25

**Authors:** Tobias Tyrberg, Lars Hagberg, Staffan Nilsson, Anna Grahn

**Affiliations:** ^1^ Department of Infectious Diseases, Institute of Biomedicine, Sahlgrenska Academy University of Gothenburg Gothenburg Sweden; ^2^ Department of Infectious Diseases Sahlgrenska University Hospital Gothenburg Sweden; ^3^ Department of Laboratory Medicine, Institute of Biomedicine, Sahlgrenska Academy University of Gothenburg Gothenburg Sweden

**Keywords:** central nervous system (CNS), encephalitis, epidemiology, immunosuppression, varicella‐zoster virus (VZV)

## Abstract

The determinants of varicella‐zoster virus (VZV)‐associated central nervous system (CNS) infection have not been fully elucidated. This study aimed to investigate the incidence and risk factors, including immunosuppression, for different manifestations of VZV‐associated CNS infection. Patient registers were used to include adults diagnosed with VZV‐associated CNS infections between 2010 and 2019 in Sweden. Nationwide registers covering specialized care, and regional registers covering primary care, were used. Controls without a VZV diagnosis during the study period were matched by age and sex. Risk factors were calculated using multivariable logistic regression. A total of 1488 adult cases with VZV‐associated CNS infection were identified, yielding an incidence of 1.92/100 000 person‐years, which increased over the study period. Meningitis was the most frequent (45%), followed by encephalitis (38%), and Ramsay Hunt syndrome (17%). The highest incidence was observed in individuals over 70 years of age (4.15/100 000 person‐years), in whom encephalitis was most common. Statistically significant risk factors for VZV‐associated CNS infection were HIV, hematological cancer, treatment with specific immunosuppressants or glucocorticoids, chronic obstructive pulmonary disease, diabetes, solid cancer, stroke, and congestive heart failure. Encephalitis was associated with older age, more immunosuppressive conditions, and more comorbidities than other manifestations. In conclusion, VZV is a common cause of adult viral CNS infection, for which elderly individuals with immunosuppressive or comorbid conditions are at the highest risk. The strongest risk factors found were HIV, hematological cancer, and treatment with specific immunosuppressants or high‐dose glucocorticoids.

## 1 Introduction

Varicella‐zoster virus (VZV) is one of the most common causes of viral central nervous system (CNS) infection [1, 2]. After a primary infection causing varicella, the virus establishes latency in neurons and reactivates most commonly as herpes zoster. Rarely, VZV reactivates to cause CNS infection, including encephalitis, vasculitis, meningitis, and peripheral facial palsy (Ramsay Hunt syndrome [RHS]), potentially leading to significant morbidity or mortality in affected individuals [3, 4]. The overall incidence of herpes zoster is estimated to be between 3 and 5 per 1000 person‐years. It has increased over the past decades in several countries, independent of changes in age and immunosuppression [5, 6, 7]. Similarly, the incidence of VZV‐associated CNS infection has reportedly also increased over time [8, 9]. However, the calculated incidence rates for VZV‐associated CNS infection are mostly based on small studies with varying results [4, 9, 10].

Risk factors for herpes zoster are well‐known and include older age, immunosuppression due to disease or treatment, and chronic diseases [11]. By contrast, the risk factors for VZV‐associated CNS infection have been insufficiently studied. Nonetheless, there are indications of the important role of innate and T‐cell immunity, as indicated by severe VZV‐associated CNS infections in certain specific immunodeficiencies [12, 13]. Furthermore, patients with hematological cancer experience more zoster episodes as well as a higher proportion of CNS‐related zoster complications, as compared with patients with solid cancer [14]. Omland et al. demonstrated that risk factors of VZV‐associated CNS infection included comorbidities and immunosuppressive conditions, but they did not investigate the contribution of different diagnoses or medications in detail [15]. Here, regional and nationwide registers were used to estimate the adult incidence of different manifestations of VZV‐associated CNS infection between 2010 and 2019. In addition, cases with VZV‐associated CNS infection were compared to matched controls without a VZV diagnosis during the study period to identify specific risk factors with a focus on immunosuppressive conditions and comorbidities.

## 2 Materials and Methods

### 2.1 Setting

This retrospective register‐based case‐control study was conducted using data from Swedish national and regional health registers between 2000 and 2019. Registered residents in Sweden are given a unique personal identity number that is used in all contacts with healthcare services, making it possible to link information about patients between the different health registers. Episodes of VZV infection were identified through such diagnosis registers. The National Patient Register (NPR) covers all specialized care, including in‐ and outpatient visits. Since no national *primary* healthcare register exists in Sweden, two regional registers which record diagnoses from primary care visits were used, namely VEGA (Västra Götaland county) and VAL (Stockholm county). These are the two largest counties in Sweden and together comprise approximately 40% of the national population. The study was approved by the Swedish Ethics Review Authority (registration numbers 2020‐03623 and 2023‐06176‐02). As it was an observational study using anonymized data, no informed consent from the study participants was deemed necessary.

### 2.2 Validation Study

The algorithms to identify cases with VZV‐associated CNS infection were validated in a separate validation study. The aim was to randomly include 40 adults cases for each of the diagnoses: encephalitis, meningitis (between 2010 and 2019), and RHS (between 2000 and 2021). International Classification of Diseases 10th Revision (ICD‐10) code combinations were used to identify cases (Supporting Information S1: Table S1). For easier access to medical records, the cases were extracted from the VEGA local regional register, which covers in‐ and outpatient care as well as primary care in the county of Västra Götaland in Sweden. One of the authors (T.T.) reviewed the medical records to validate the diagnoses using the predefined criteria (stated in the following paragraph). Ambiguous cases were discussed within the research team.

### 2.3 Case Definitions

The clinical diagnosis of encephalitis is based on a combination of clinical findings: cerebrospinal fluid (CSF) pleocytosis, microbiological evidence of a typical pathogen, and electroencephalographical and neuroimaging abnormalities. While different sets of criteria have been proposed, they typically include one or more of the following major criteria: altered mental status, focal neurological findings, and seizures. Since 2013, the most established criteria are those of the International Encephalitis Consortium, with altered mental status as a mandatory finding [16]. To avoid exclusion of true encephalitis cases, two additional sets of criteria were used, which also include cases with signs of focal or multifocal neurological deficits such as dysphasia or motor weakness, even without altered mental status [17, 18]. If a patient fulfilled the criteria for at least one of the three sets of criteria, the patient was considered as having encephalitis. Meningitis was defined as patients with CSF pleocytosis, no signs of parenchymatous brain dysfunction, and presenting with clinical signs of meningitis. RHS cases were required to exhibit peripheral facial nerve palsy and signs of VZV as a cause (either from typical blisters in the ear or mouth, or virological confirmation from blisters or CSF). Positive VZV polymerase chain reaction (PCR) in CSF or positive VZV immunoglobulin G‐index at the time of diagnosis was mandatory to meet the diagnostic criteria for encephalitis and meningitis. (Details of definitions are presented in Supporting Information S1: Table S2).

### 2.4 Study Population

For the register‐based case‐control study, all patients with at least one registered VZV infection (ICD‐10 code B01 or B02) between January 1, 2010 and December 31, 2019 were identified. All adults (≥ 18 years old) with at least one episode of VZV‐associated CNS infection were included as cases in this study. VZV‐associated CNS infection was defined as having received one of the following ICD‐10 diagnostic codes: B01.0 or B02.1 (meningitis), B01.1 or B02.0 (encephalitis), or either B01.8 or B02.8 combined with one of G94.8 (cerebellitis), G51.0 (RHS), or I77.8 (vasculitis; Supporting Information S1: Table S3). The date of the first such diagnosis was called the index date. Diagnoses of RHS made exclusively in primary healthcare were included, but all other VZV‐associated CNS diagnoses were only counted if diagnosed in specialized care, as they require a lumbar puncture for diagnosis. Cases with both a diagnosis of encephalitis and meningitis or RHS were only counted as cases of encephalitis. Controls, matched by age, sex, and place of residence, but without a VZV diagnosis during the study period, were identified through a population register (Statistics Sweden) in a 3:1 ratio, and received the same index date as the matching case.

### 2.5 Clinical Data

Comorbidities, including immunosuppressive conditions, were defined based on ICD‐10 codes and drugs using Anatomical Therapeutic Chemical (ATC) codes. To include chronic conditions, ICD‐10 codes from the year 2000 onward were retrieved. For cases and matched controls identified through the regional primary care registers, diagnoses were first extracted from the respective register of inclusion, then updated with data from the NPR, as applicable. ATC codes were extracted from the National Prescribed Drug Register, which contains nationwide data on all prescribed drugs from the year 2005 onward. To more accurately include current comorbidities, time frames were applied according to the chronicity of the respective disease, as adapted from Hvidberg et al. [19]. Details on the definitions of comorbidities and drugs are outlined in Supporting Information S1: Tables S4–S6.

### 2.6 Statistical Analyses

For the positive predictive values (PPVs), binomial proportion confidence intervals (CIs) were calculated. Incidence per 100 000 person‐years was calculated using the mid‐year adult Swedish population between 2010 and 2019 as the denominator (public data from Statistics Sweden). For RHS cases exclusively identified in the regional primary care registers, the regional population was used as the denominator. Each VZV‐associated CNS infection was only counted once per study person within the study period. Demographic data are presented as medians, interquartile ranges, and percentages. The change in incidence rate over the study period was evaluated using the chi‐square test for trend. To evaluate risk factors, odds ratios (OR) were calculated using both univariable and multivariable conditional logistic regression, with the matched sets as strata. The comorbidities and treatments assumed to be linked with VZV‐associated CNS infection were selected based on previous data [11, 13, 20]. The following variables were selected and adjusted for in the multivariable model: HIV, solid cancer, hematological cancer, transplantation, primary immunodeficiency, diabetes, chronic obstructive pulmonary disease (COPD), alcohol abuse, depression, glucocorticoids, antineoplastic agents, and immunosuppressants. Further comorbidities were included only if significant, using forward stepwise selection. A *p* < 0.05 was considered statistically significant and 95% CI were used. IBM SPSS Statistics, version 29.0 (IBM Corp., Armonk, NY, USA) and R, version 4.2.2 were used for data processing and statistical analyses.

## 3 Results

### 3.1 Case Validation

To validate VZV‐associated CNS infection diagnoses the medical records of 114 subjects were reviewed, of which 40 were diagnosed as encephalitis, 40 as meningitis, and 34 as RHS. Two‐thirds of the validated cases had received an ICD‐10 code for zoster‐related complications (B02) and the remainder were diagnosed with varicella‐related complications (B01; Supporting Information S1: Table S1). The PPV for meeting the predefined criteria was 73%, 58%, and 88% for encephalitis, meningitis, and RHS, respectively. Some cases not meeting the criteria were considered probable after review of the records (encephalitis *n* = 2; meningitis *n* = 1). Including these probable cases, the PPV reached 78% (encephalitis), 60% (meningitis), and 88% (RHS), while the PPVs for having any VZV‐associated CNS infection when diagnosed with encephalitis, meningitis, or RHS were 93%, 85%, and 88%, respectively (Supporting Information S1: Table S7). The overall PPV was 89% (95% CI [82–94]) for having any VZV‐associated CNS infection when diagnosed as either encephalitis, meningitis, or RHS. Further details of the results of the validation study are found in Supporting Information S1: Tables S8–S11.

### 3.2 Case Identification and Baseline Characteristics

A total of 1488 individuals ≥ 18 years of age received at least one diagnosis of VZV‐associated CNS infection between 2010 and 2019, and 3823 controls were matched regarding age, sex, and place of residence. The cases were diagnosed as encephalitis (*n* = 571), meningitis (*n* = 669), RHS (*n* = 252), or vasculitis (*n* = 1). An ICD‐10 code for zoster‐related complications (B02) was used in 73% of encephalitis cases, 64% of meningitis cases, and 90% of RHS cases (Supporting Information S1: Table S3). Five individuals who were diagnosed as having meningitis and RHS were counted in both groups. No cases of cerebellitis were identified. Four cases with RHS were identified exclusively in primary healthcare. Cases with VZV‐associated CNS infection had a median age of 61 (interquartile range [IQR] 37–76 years), with encephalitis, rather than meningitis, generally affecting older patients (median 75 vs. 45 years; Table 1). The ratio between females and males was close to 1:1 in all groups. Encephalitis cases had more comorbid conditions, including immunosuppressive diseases, than cases with other manifestations. The proportion of patients treated with any immunosuppressive drug was similar for the different groups, that is, cases with encephalitis (21.5%), meningitis (17.0%), and RHS (16.7%). Details about immunosuppressive diseases and drugs included are listed in Supporting Information S1: Tables S4 and S5.

**Table 1:** Demographic data at VZV diagnosis for cases and controls.

	VZV‐associated CNS infection		Encephalitis		Meningitis		Ramsay Hunt syndrome	
Variable, median (IQR) or *n* (%)	Cases (*n* = 1488)	Controls (*n* = 3823)	Cases (*n* = 571)	Controls (*n* = 1399)	Cases (*n* = 669)	Controls (*n* = 1701)	Cases (*n* = 252)	Controls (*n* = 735)
Age	61 (37–76)	61 (37–76)	75 (59–82)	75 (59–82)	45 (31–65)	45 (31–65)	62 (46–72)	62 (46–73)
Sex, female	742 (49.9)	1,890 (49.4)	276 (48.3)	669 (47.8)	337 (50.4)	842 (49.5)	130 (51.6)	382 (52.0)
Immunosuppressive condition^a^	390 (26.2)	445 (11.6)	189 (33.1)	217 (15.5)	149 (22.3)	136 (8.0)	54 (21.4)	94 (12.8)
Immunosuppressive disease	194 (13)	224 (5.9)	113 (19.8)	120 (8.6)	63 (9.4)	57 (3.4)	20 (7.9)	50 (6.8)
HIV	9 (0.6)	3 (0.1)	5 (0.9)	2 (0.1)	4 (0.6)	1 (0.1)	0 (0)	0 (0)
Solid cancer	124 (8.3)	198 (5.2)	74 (13)	107 (7.6)	38 (5.7)	47 (2.8)	12 (4.8)	47 (6.4)
Hematological cancer	58 (3.9)	26 (0.7)	37 (6.5)	13 (0.9)	16 (2.4)	10 (0.6)	6 (2.4)	3 (0.4)
Transplantation	23 (1.5)	4 (0.1)	12 (2.1)	0 (0)	9 (1.3)	2 (0.1)	3 (1.2)	2 (0.3)
Primary immunodeficiency	6 (0.4)	2 (0.1)	2 (0.4)	1 (0.1)	2 (0.3)	0 (0)	2 (0.8)	1 (0.1)
Immunosuppressive drug	279 (18.8)	259 (6.8)	124 (21.7)	117 (8.4)	114 (17)	86 (5.1)	42 (16.7)	55 (7.5)
Glucocorticoids	237 (15.9)	217 (5.7)	109 (19.1)	101 (7.2)	97 (14.5)	70 (4.1)	31 (12.3)	45 (6.1)
High‐dose^b^	28 (1.9)	9 (0.2)	10 (1.8)	4 (0.3)	14 (2.1)	2 (0.1)	5 (2.0)	3 (0.4)
Antineoplastic agents	25 (1.7)	15 (0.4)	14 (2.5)	7 (0.5)	9 (1.3)	4 (0.2)	2 (0.8)	4 (0.5)
Cyclophosphamide	5 (0.3)	0 (0)	4 (0.7)	0 (0)	1 (0.1)	0 (0)	0 (0)	0 (0)
Immunosuppressants	105 (7.1)	45 (1.2)	43 (7.5)	14 (1.0)	45 (6.7)	18 (1.1)	18 (7.1)	13 (1.8)
Azathioprine	25 (1.7)	3 (0.1)	10 (1.8)	0 (0)	9 (1.3)	1 (0.1)	6 (2.4)	2 (0.3)
Ciclosporin	8 (0.5)	1 (0)	2 (0.4)	0 (0)	5 (0.7)	0 (0)	1 (0.4)	1 (0.1)
TNF‐α inhibitors	16 (1.1)	6 (0.2)	4 (0.7)	0 (0)	10 (1.5)	3 (0.2)	2 (0.8)	3 (0.4)
JAK inhibitors	5 (0.3)	0 (0)	3 (0.5)	0 (0)	1 (0.1)	0 (0)	1 (0.4)	0 (0)
Diabetes	183 (12.3)	275 (7.2)	107 (18.7)	138 (9.9)	41 (6.1)	89 (5.2)	35 (13.9)	48 (6.5)
COPD	70 (4.7)	71 (1.9)	45 (7.9)	31 (2.2)	12 (1.8)	23 (1.4)	12 (4.8)	17 (2.3)
Alcohol abuse	30 (2.0)	52 (1.4)	12 (2.1)	18 (1.3)	13 (1.9)	24 (1.4)	5 (2.0)	10 (1.4)
Depression	60 (4.0)	102 (2.7)	29 (5.1)	43 (3.1)	25 (3.7)	41 (2.4)	6 (2.4)	18 (2.4)
Stroke	72 (4.8)	120 (3.1)	46 (8.1)	75 (5.4)	15 (2.2)	22 (1.3)	12 (4.8)	23 (3.1)
Ischemic heart disease	42 (2.8)	78 (2.0)	27 (4.7)	51 (3.6)	10 (1.5)	20 (1.2)	5 (2.0)	7 (1.0)
Congestive heart failure	105 (7.1)	153 (4.0)	69 (12.1)	101 (7.2)	21 (3.1)	29 (1.7)	14 (5.6)	23 (3.1)
Asthma	70 (4.7)	100 (2.6)	29 (5.1)	45 (3.2)	34 (5.1)	36 (2.1)	6 (2.4)	20 (2.7)
Valaciclovir^c^	905 (60.8)	3 (0.1)	262 (45.9)	1 (0.1)	466 (69.7)	1 (0.1)	182 (72.2)	1 (0.1)
Aciclovir^c^	129 (8.7)	1 (0)	54 (9.5)	1 (0.1)	48 (7.2)	0 (0)	27 (10.7)	0 (0)

Abbreviations: CNS, central nervous system; COPD, chronic obstructive pulmonary disease; HIV, human immunodeficiency virus; IQR, interquartile range; TNF‐α, tumor necrosis factor alpha; VZV, varicella‐zoster virus.

^a^ Any immunosuppressive disease or drug.

^b^ High‐dose defined as average daily dose > 10 mg prednisolone equivalents, regardless of age and weight.

^c^ Only prescribed drugs are presented, as data on inpatient treatment is not available.

### 3.3 Incidence

**Figure 1:**
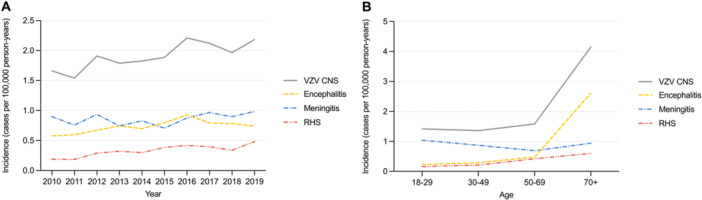
Line plots showing incidences of VZV‐associated CNS infections per 100 000 person‐years in adults (≥ 18 years old). (A) Yearly incidences of VZV‐associated CNS infections 2010–2019. (B) Average incidences in different age strata: ages 18–29, 30–49, 50–69, and 70 and older. Four cases with RHS were identified exclusively by primary healthcare. Primary healthcare registers cover 39% of total population, therefore four cases represent 4/0.39 ≈ 10 cases for the calculation of national incidence. CNS, central nervous system; RHS, Ramsay Hunt syndrome; VZV, varicella‐zoster virus.

The average incidence of VZV‐associated CNS infection was 1.92/100 000 person‐years in the adult population. For the different manifestations, the average incidences were 0.73/100 000 person‐years for encephalitis, 0.86/100 000 person‐years for meningitis, and 0.33/100 000 person‐years for RHS (Figure 1A). The yearly incidence rate of VZV‐associated CNS infection increased between 2010 and 2019 (*p* < 0.001). The highest incidence of VZV‐associated CNS infection was observed in individuals over 70 years of age, with 4.15 cases per 100 000 person‐years (Figure 1B). This increase consisted mostly of encephalitis cases, which accounted for 63% of the incidence in individuals ≥ 70 years, while meningitis accounted for 73% in the age group 18–29 years. RHS was more uncommon, accounting for approximately 17% of the total incidence of VZV‐associated CNS infection. Both encephalitis and RHS incidence increased with advancing age.

### 3.4 Risk Factors for VZV‐Associated CNS Infection

To identify risk factors for VZV‐associated CNS infection, adjusted OR for different comorbid conditions and treatments were calculated. Statistically significant adjusted OR for VZV‐associated CNS infection compared to controls were as follows: for HIV 6.0 (95% CI: 1.6–23.1), hematological cancer 3.4 (95% CI: 2.1–5.8), treatment with specific immunosuppressants 3.7 (95% CI: 2.4–5.5), high‐dose glucocorticoids 4.3 (95% CI: 1.9–9.7), low or unknown dose glucocorticoids 2.0 (95% CI: 1.6–2.6), COPD 1.9 (95% CI: 1.3–2.8), diabetes 1.7 (95% CI: 1.4–2.2), solid cancer 1.4 (95% CI: 1.0–1.8), stroke 1.4 (95% CI: 1.0–2.0), congestive heart failure 1.4 (95% CI: 1.0–1.9; Table 2). Comparison of the different manifestations of VZV‐associated CNS infection—encephalitis, meningitis, and RHS—indicated two common risk factors observed in all groups independently: hematological cancer and treatment with immunosuppressants. By contrast, COPD was a risk factor for encephalitis, but not for meningitis or RHS.

**Table 2:** Risk factors of VZV‐associated CNS infection.

	VZV‐associated CNS infection		Encephalitis		Meningitis		Ramsay Hunt syndrome	
Variable	Univariable OR (95% CI)	Adjusted OR^a^ (95% CI)	Univariable OR (95% CI)	Adjusted OR^a^ (95% CI)	Univariable OR (95% CI)	Adjusted OR^a^ (95% CI)	Univariable OR (95% CI)	Adjusted OR^a^ (95% CI)
HIV	7.7 (2.1–28.5)	**6.0 (1.6–23.1)**	6.0 (1.2–31.1)	4.9 (0.9–27.1)	11.2 (1.2–100)	7.6 (0.8–73.6)	*n/a*	*n/a*
Solid cancer	1.7 (1.3–2.2)	**1.4 (1.0–1.8)**	1.8 (1.3–2.6)	**1.5 (1.0–2.1)**	2.2 (1.4–3.6)	**2.3 (1.4–3.8)**	0.7 (0.4–1.4)	0.5 (0.2–1.1)
Hematological cancer	5.4 (3.4–8.6)	**3.4 (2.1–5.8)**	6.5 (3.5–12.4)	**3.7 (1.8–7.7)**	4.1 (1.8–9.0)	**2.8 (1.1–7.1)**	6.0 (1.5–24.0)	**4.9 (1.1–22.2)**
Transplantation	14.7 (5.1–42.9)	2.0 (0.6–6.4)	*n/a*	*n/a*	11.6 (2.5–54.5)	2.0 (0.4–11.0)	4.5 (0.8–26.9)	0.5 (0.1–5.1)
Primary immunodeficiency	8.2 (1.6–40.8)	5.9 (0.9–37.6)	4.6 (0.4–51.3)	7.2 (0.3–157)	*n/a*	*n/a*	6.0 (0.5–66.2)	7.6 (0.5–114)
Diabetes	1.9 (1.6–2.4)	**1.7 (1.4–2.2)**	2.2 (1.7–2.9)	**2.1 (1.5–2.9)**	1.2 (0.8–1.8)	1.0 (0.7–1.6)	2.5 (1.5–4.1)	**2.7 (1.6–4.6)**
COPD	2.6 (1.8–3.7)	**1.9 (1.3–2.8)**	3.7 (2.3–6.0)	**3.1 (1.9–5.3)**	1.3 (0.7–2.7)	0.7 (0.3–1.7)	2.2 (1.0–4.9)	1.8 (0.8–4.2)
Alcohol abuse	1.5 (0.96–2.4)	1.2 (0.7–2.0)	1.7 (0.8–3.5)	1.2 (0.5–2.7)	1.4 (0.7–2.7)	1.2 (0.6–2.5)	1.5 (0.5–4.5)	2.0 (0.6–6.8)
Depression	1.5 (1.1–2.1)	1.2 (0.8–1.7)	1.7 (1.0–2.8)	1.5 (0.9–2.5)	1.6 (0.96–2.7)	1.4 (0.8–2.4)	0.99 (0.4–2.5)	0.5 (0.2–1.5)
Immunosuppressive drug	3.2 (2.7–3.9)	b	3.0 (2.2–3.9)	b	4.1 (3.0–5.6)	b	2.5 (1.6–3.9)	b
Glucocorticoids	3.2 (2.6–3.9)	b	2.9 (2.2–3.9)	b	4.2 (3.0–5.9)	b	2.3 (1.4–3.7)	b
High‐dose^c^	7.9 (3.7–16.8)	**4.3 (1.9–9.7)**	6.0 (1.9–19.2)	3.2 (0.9–11.8)	17.9 (4.0–78.8)	**14.0 (2.9–67.8)**	4.8 (1.1–20.0)	2.9 (0.5–17.7)
Low or unknown dose	2.9 (2.3–3.5)	**2.0 (1.6–2.6)**	2.8 (2.1–3.8)	**1.9 (1.3–2.6)**	3.5 (2.5–5.0)	**2.6 (1.8–3.9)**	2.0 (1.2–3.4)	1.6 (0.9–3.0)
Antineoplastic agents	4.2 (2.2–8.0)	1.9 (0.9–4.0)	5.1 (2.0–12.6)	2.0 (0.7–6.1)	5.1 (1.6–16.7)	3.4 (0.9–13.0)	1.5 (0.3–8.2)	0.8 (0.1–5.9)
Immunosuppressants	6.3 (4.4–9.0)	**3.7 (2.4–5.5)**	7.9 (4.3–14.8)	**4.9 (2.4–9.8)**	6.6 (3.8–11.6)	**3.3 (1.7–6.2)**	4.2 (2.0–8.7)	**3.9 (1.6–9.3)**
Stroke	1.6 (1.2–2.2)	**1.4 (1.0–2.0)**	1.6 (1.1–2.4)	—	1.7 (0.9–3.4)	—	1.6 (0.8–3.3)	—
Ischemic heart disease	1.4 (0.94–2.0)	—	1.3 (0.8–2.1)	—	1.3 (0.6–2.8)	—	2.1 (0.7–6.8)	—
Congestive heart failure	1.9 (1.4–2.5)	**1.4 (1.0–1.9)**	1.8 (1.3–2.5)	—	2.0 (1.1–3.8)	—	1.8 (0.9–3.6)	—
Asthma	1.8 (1.3–2.5)	—	1.6 (0.97–2.6)	—	2.5 (1.5–4.0)	—	0.9 (0.3–2.2)	—

*Note:* Statistically significant adjusted odds ratios (*p* < 0.05) are marked in bold. Abbreviations: CI, confidence interval; COPD, chronic obstructive pulmonary disease; HIV, human immunodeficiency virus; n/a, not applicable; OR, odds ratio; VZV, varicella‐zoster virus.

^a^ Variables included for the multivariable model: HIV, solid cancer, hematological cancer, transplantation, primary immunodeficiency, diabetes, COPD, alcohol abuse, depression, glucocorticoids, antineoplastic agents, and immunosuppressants. Further variables were entered with a forward stepwise selection and dropped when not significant (marked “—” in the table).

b Only subgroups included in multivariable model.

^c^ High‐dose defined as average daily dose > 10 mg prednisolone equivalents, regardless of age and weight.

## 4 Discussion

This nationwide Swedish study confirms that VZV is an important cause of viral CNS infections, with a calculated adult incidence of 1.92/100 000 person‐years. Its highest incidence was found in individuals over 70 years of age. Retrospective data from registers were used to identify one of the largest cohorts of VZV‐associated CNS infection ever assembled, making it possible to detect a wide range of risk factors.

The calculated incidence agrees with a previous estimation of 1.8/100 000 person‐years, based on positive VZV DNA in the CSF in a small regional study in Sweden [4]. A UK study from 2004 to 2013 with a large VZV cohort yielded a lower incidence of 0.45/100 000 person‐years, but the incidence increased rapidly over the study period, suggesting significant changes in diagnostics and laboratory reporting at the time [9]. In the present study, the incidence of VZV‐associated CNS infection also increased, although not to the same extent. Similarly, a recent study in France showed an increased incidence of VZV encephalitis between 2007 and 2019 [8]. Indeed, the incidence of herpes zoster has increased irrespective of immunosuppression and age, and in both vaccination and non‐vaccination countries [5, 6, 7]. On the other hand, during the 10‐year study period, there was an increase in both the average age in Sweden and the proportion of those with immunosuppression in all controls (data not shown), which may partly explain the increased incidence rate of VZV‐associated CNS infections.

The most common manifestation of VZV‐associated CNS infection was meningitis, followed by encephalitis. Two large prospective studies in Denmark have recently published incidence rates of VZV encephalitis (0.53/100 000 person‐years) and VZV meningitis (0.7/100 000 person‐years) [21, 22]. These are figures which are slightly lower than those in the present study. While their stricter inclusion criteria may have missed some cases, the present study is at higher risk of false positive cases, especially in the meningitis group, which showed a low diagnosis validity. Encephalitis was the dominating CNS manifestation in individuals over 70 years of age, which is in line with an earlier Spanish study [23]. Former studies have reported both higher and lower RHS incidence than the present study, but the various inclusion criteria make comparisons difficult [24, 25]. The inclusion of cases from primary care registers did not notably affect the calculated incidence, which may be due to diagnostic difficulties when relying solely on clinical criteria [26]. It is, therefore, probable that RHS incidence was underestimated due to unidentified cases. Only one patient was diagnosed with vasculitis, although this is a well‐known complication in VZV infection [27]. This may have resulted from underdiagnosis or incorrect coding, but as this complication is rare, it is difficult to detect an incorrect diagnosis in a validation study with only a limited number of cases.

Independent risk factors for VZV‐associated CNS infection were HIV, solid or hematological cancer, treatment with glucocorticoids and specific immunosuppressants, COPD, diabetes, stroke, and congestive heart failure. The findings of the present study highlight the link between immunosuppressive conditions and the risk of severe herpes zoster manifestations. Cell‐mediated and innate immunity play an important role in the host defense against VZV infection and reactivation [11]. Immunosuppressive diseases that involve an increased risk of VZV‐associated CNS infection were HIV and cancer. The last named constitutes a large group with different levels of immunosuppression, and has been shown to be overrepresented in VZV‐associated CNS infection [15]. HIV and hematological cancer affect the cell‐mediated immune system and have earlier been linked to both peripheral and CNS manifestations caused by VZV [11, 13].

Transplantation and primary immunodeficiency did not independently predict a higher risk of VZV‐associated CNS infection. However, transplantation is a well‐established risk factor for herpes zoster, including CNS complications [11, 13]. The results of the present study indicate that the heightened risk may be attributable to the ongoing treatment with specific immunosuppressants or glucocorticoids, something that is common in this patient group, rather than the transplantation diagnosis. The risk of herpes zoster is increased in several primary immunodeficiencies [13], some also being linked to a considerable risk of CNS infection [12]. However, primary immunodeficiencies are diverse and were rare in the present study, which factor presumably contributed to the lack of significant correlation with VZV‐associated CNS infection.

Specific immunosuppressants and corticosteroid treatment were risk factors for VZV‐associated CNS infection. Immunosuppressive treatment is becoming increasingly prevalent across different medical specialties, potentially heightening the future risk of complications caused by VZV. Specific immunosuppressants constitute a heterogeneous group, including many of the medications proposed as risk factors for VZV‐associated CNS infection such as JAK inhibitors, anti‐α4β1‐integrin antibodies, and sphingosine‐1‐phosphate‐receptor‐modulator [13]. Corticosteroids affect the immune system extensively, including T‐cells, B‐cells, and neutrophils. Their administration has been shown to induce lymphopenia, leading to less release of cytokines and interferons, which could explain the heightened risk of herpes zoster [13, 28, 29]. Corticosteroids have also been reported as a risk factor in vaccine strain varicella meningitis [30]. Higher doses were related to an increased risk of VZV‐associated CNS infection, in agreement with studies on herpes zoster [31, 32]. In addition, antineoplastic agents have also been linked to an increased risk of herpes zoster [28], but did not appear as independent risk factors for VZV‐associated CNS infection in the present study cohort. On the other hand, this may be due to overlap with cancer diagnoses, which were detected as significant risk factors. Furthermore, since the available data included prescribed treatments only, it has not been possible to investigate an association between hospital‐based medication and VZV‐associated CNS infection.

Older age is the strongest risk factor for herpes zoster [11], which agrees with the association between advancing age and the higher incidence of encephalitis and RHS seen in this study. By contrast, meningitis incidence was stable across different age strata and generally affected younger individuals, as shown in other VZV CNS cohorts [4, 33]. Also, in vaccine strain VZV CNS infection, the majority of such children or young adolescents are diagnosed with VZV meningitis, independent of immunestatus [30, 34]. Furthermore, in the present study, individuals with meningitis had fewer comorbidities, as compared to their age‐matched controls. The reason predominantly younger people suffer from meningitis and older ones from encephalitis is not known. Perhaps an aging immune system, or other factors including comorbidities, increase the risk of the virus reaching the brain, rather than only staying in the meninges [13].

Several comorbidities, other than directly immunosuppressive disorders, were overrepresented in cases with VZV‐associated CNS infection. Four were significant risk factors in the multivariable analysis: diabetes, COPD, stroke, and congestive heart failure. Patients with diabetes are susceptible to infections due to multiple factors, including diminished cell‐mediated immune response [35], but studies on the risk of herpes zoster have shown conflicting results [28, 36, 37]. COPD has also been found to be a risk factor for herpes zoster and it was overrepresented in an earlier VZV CNS cohort [15, 38]. Furthermore, it has been proposed that the innate immune system of patients with COPD becomes exhausted through the course of disease progression [39], creating a possible explanation to the heightened risk of herpes zoster. VZV is able to induce a vasculopathy leading to stroke [27], but here earlier stroke appeared as an independent risk factor for VZV‐associated CNS infection. However, since diagnoses up until index date were included, some cases may in fact be incident stroke caused by the VZV infection. Although there might be an unknown additive effect of multiple comorbidities increasing the overall risk of VZV‐associated CNS infection, the findings could be influenced by information bias. Patients with COPD and congestive heart failure are mainly followed in the primary healthcare system, which is not included in the NPR. The probability of such diagnoses being recorded in the NPR depends on a patient being referred for specialized care. Since almost all cases with VZV‐associated CNS infection are, in fact, seen in specialized care, this could skew the result toward higher OR.

All CNS manifestations affected males and females equally, in contrast to herpes zoster, which has a female predilection [20]. One plausible reason may be the differences in health‐seeking behavior between men and women, although this would be expected to have less influence in individuals suffering from the more severe symptoms of VZV‐associated CNS infection.

To the best of our knowledge, this is the first study to validate ICD‐10 code diagnoses of VZV‐associated CNS infections, and only a few studies have validated diagnoses for other CNS infections. The ICD‐10 code B00.4 for herpes simplex encephalitis only yielded a PPV of 58%, but in combination with G05.1E it reached 76% [40]. Henriksen et al. found a PPV of 64% for different types of community‐acquired CNS infections, and a decreasing PPV in cases with more severe disease and greater comorbidities [41]. In the present study, encephalitis is the most complex and severe of the diagnoses included, and yet it reached a validity of 73% based on the predefined criteria. The method of including three sets of criteria probably contributed to a fairly high PPV. On the other hand, the validity of the meningitis diagnosis was poor. A possible explanation could be that the threshold for a meningitis diagnosis is lower in uncertain cases than for encephalitis. Furthermore, the clinical manifestations of VZV‐associated CNS infections occasionally overlap in the same individual. Indeed, a high proportion (89%) of the validated cases were considered to have a VZV‐associated CNS infection, although not diagnosed with the correct, that is, specific, VZV CNS diagnosis. ICD‐10 codes for both varicella and zoster‐related complications were used. However, it is likely that the majority of VZV cases in this adult population suffered from a reactivated disease, since the VZV seroprevalence of Swedish 12‐year‐olds is as high as 92% [42].

Despite the strength of a nationwide cohort, there are several limitations to this study. The first is its register‐based design that increases the risk of false positive cases. This was evident for meningitis as the least valid diagnosis. However, the overall validity of VZV‐associated CNS infection diagnoses was high, rendering calculations concerning the whole group reliable. Secondly, the NPR did not include primary care, introducing a possible information bias. Diagnoses such as COPD and congestive heart failure, mainly followed in primary care, would go unnoticed in healthier individuals without registered visits to specialized care. This bias should be less pronounced in the case of more severe diagnoses that are treated in specialized care, but would not apply for the National Prescribed Drug Register, as it covers all healthcare. Thirdly, only prescribed drugs could be identified, preventing us from examining medications dispensed in‐hospital as risk factors. Lastly, no data on vaccination status were available in the registers, although thus far the vaccination coverage remains relatively low in Sweden.

## 5 Conclusion

VZV is demonstrably an important cause of viral CNS infections. Elderly individuals are at especially high risk and more likely to present with the more severe CNS manifestation encephalitis. Several independent risk factors for VZV‐associated CNS infection have been identified, including HIV, solid or hematological cancer, treatment with specific immunosuppressants or glucocorticoids, COPD, and diabetes.

## Author Contributions

A.G., S.N., and T.T. designed the research. T.T. performed the validation. A.G. and T.T. collected data from registers. S.N. and T.T. prepared and analyzed data. T.T. wrote the first draft of the manuscript. All authors participated in interpretation of data, review of the manuscript, and approval of the final version to be submitted.

## Conflicts of Interest

GlaxoSmithKline Biologicals SA provided funding and advisory support in this collaboration study, but the authors are fully responsible for the design, data collection, analyses, and interpretation of results. LH has given lectures at GlaxoSmithKline, Pfizer, and Bavarian Nordic. The other authors declare no conflict of interests.

## Supporting information


**Appendix A. Supplementary material.** Supplementary data associated with this article is found in the attached “Supplementary material” file.

## Data Availability

The datasets used and analyzed during the current investigation are available from the corresponding author upon request.
