# Supplementary material for: Incidence and Risk Factors for Varicella‐Zoster Virus‐Associated Central Nervous System Infections: A Nationwide Swedish Retrospective Case‐Control Study
Source: J Med Virol. 2025 Jan 25;97(2):e70166. doi: 10.1002/jmv.70166 (PMC11771675; doi:10.1002/jmv.70166)
Supplement: Supplementary file 1 — Appendix A. Supplementary material. Supplementary data associated with this article is found in the attached “Supplementary material” file. [file JMV-97-e70166-s001.docx]

**Supplementary material**

[Validation study – methodology 2](#_Toc181390767)

[Supplementary Table 1. ICD-10 codes of the validation study. 3](#_Toc181390768)

[Supplementary Table 2. Definitions of VZV-associated CNS diagnoses. 4](file:////Users/tobias/Documents/Forskning/VZV+epidemiologi/Skrivandet/241005%20Revision%20JMV/Supplementary%20VZV%20epi%20rev%20linguist%20241031.docx#_Toc181390769)

[Supplementary Table 3. VZV-associated CNS diagnoses in register-based study. 6](#_Toc181390770)

[Supplementary Table 4. Definitions of comorbidities. 7](#_Toc181390771)

[Supplementary Table 5. Immunosuppressive drugs. 8](#_Toc181390772)

[Supplementary Table 6. Antiviral drugs. 9](#_Toc181390773)

[Supplementary Table 7. Positive predictive values for VZV-associated CNS infection ICD-10 codes 10](#_Toc181390774)

[Supplementary Table 8. Probable encephalitis. 11](#_Toc181390775)

[Supplementary Table 9. Encephalitis criteria fulfilment for three sets of criteria 12](#_Toc181390776)

[Supplementary Table 10. Probable meningitis. 13](#_Toc181390777)

[Supplementary Table 11. Description of cases with RHS diagnosis made exclusively in the primary healthcare sector 14](#_Toc181390778)

# Validation study – methodology

Adult cases (≥ 18 years of age) diagnosed with any of the ICD-10 codes defined in Supplementary Table 1 were requested from the regional diagnosis register in Västra Götaland (VEGA). For encephalitis and meningitis all adult cases between 2010 and 2019 were requested from the registers. The first 40 diagnoses of encephalitis and meningitis were included for diagnosis validation. The diagnosis of Ramsay Hunt syndrome (RHS) was more uncommon. The validation included cases between 2000 and 2021 (28/34 [82%] were diagnosed in 2010 and later). One of the ICD-10 code combinations for RHS (G530+B022) was found to be used for postherpetic neuralgia and therefore excluded from the study. Of the 38 remaining RHS cases identified, 34 were included (exclusions were due to inaccessible records [n = 3], and duplications [n = 1]). One of the authors (TT) reviewed the medical records to validate the diagnoses using predefined criteria (Supplementary Table 2). A positive VZV PCR in cerebrospinal fluid (and/or positive VZV IgG-index) at the time of diagnosis was mandatory to fulfil the criteria for meningitis and encephalitis, while a clinical diagnosis was sufficient for RHS.

Supplementary Table 1. ICD-10 codes of the validation study. Code combinations used to define VZV-associated CNS diagnoses validated in the study

| VZV-associated CNS diagnosis | ICD-10 codes | Cases validated (n) |
| --- | --- | --- |
| Encephalitis | B011 | 15 |
| Encephalitis | B020 | 25 |
| Meningitis | B010 | 20 |
| Meningitis | B021 | 20 |
| Ramsay Hunt syndrome | B018+G510 | 3* |
| Ramsay Hunt syndrome | B028+G510 | 29* |

Abbreviations: ICD-10, International Classification of Diseases 10th Revision; VZV, varicella-zoster virus

* 2 cases with other B-diagnosis + G510

| **Diagnosis**  Supplementary Table 2. Definitions of VZV-associated CNS diagnoses. Definitions used to validate the ICD-10 code combinations specified in Supplementary Table 1 | **Mandatory findings*** | **Other clinical findings** | **CSF** | **Neuroimaging** | **EEG** | **Definition** | **Ref** |
| --- | --- | --- | --- | --- | --- | --- | --- |
| Encephalitis  Alternative 1 | Altered mental status (at least **one** of the following):  1. decreased or altered level of  consciousness and/or  2. lethargy and/or  3. personality change  lasting ≥ 24 h with no alternative cause  identified | a. Fever ≥ 38°C within 72 h before or after  presentation  b. Seizures  c. (New) focal neurological findings | Pleocytosis  (WBC > 4/mm3) | Abnormality suggestive of encephalitis | Abnormality consistent with encephalitis (not attributable to other cause) | Probable/confirmed: Mandatory findings **and** ≥ 3 “minor criteria” (i.e., Other clinical findings + CSF + neuroimaging + EEG).  [Confirmed: Requires 1. Pathologic confirmation, **or** 2. Microbiological evidence of acute infection with microorganism strongly associated with encephalitis.]  Possible: Mandatory findings **and** 2 “minor criteria” | ^1^ |
| Meningitis | 1. CSF pleocytosis (> 4 /mm3)  2. No acute signs of parenchymatous brain dysfunction | a. Headache  b. Nausea/vomiting  c. Light sensitivity  d. Neck stiffness  e. Fever > 38°C | 1. CSF pleocytosis (> 4 /mm3)  2. Negative bacterial culture from CSF | - | - | Confirmed diagnosis:  Mandatory findings  **and**  at least 2 of “other clinical findings”  Probable diagnosis:  i. As confirmed diagnosis, but only 1 of criteria a–d fulfilled. (Only e [fever] is not sufficient) | ^2^ |
| Ramsay Hunt syndrome | Peripheral facial nerve palsy  and positive VZV DNA in CSF **or** from blisters | skin blisters (ipsilateral ear, on the hard palate, or on the anterior two thirds of the tongue) | - | - | - | Confirmed diagnosis:  Peripheral facial nerve palsy  **and** Positive VZV PCR in CSF or from blister fluid (and/or positive VZV IgG-index in CSF sample)  Probable diagnosis:  Peripheral facial nerve palsy  **and** herpes zoster blisters on ipsilateral ear, palate or tongue | ^2^ |
| *A positive VZV PCR in cerebrospinal fluid (and/or positive VZV IgG-index) at the time of diagnosis was mandatory to fulfil the criteria for meningitis and encephalitis |  |  |  |  |  |  |  |
| Encephalitis  Alternative 2 | Encephalopathy (presence of a and b):  a. Depressed or altered level of  consciousness, lethargy, or  personality change lasting > 24 hours  b. One or more of the following  i. Decreased or absent response to  environment  ii. Decreased or absent eye contact  iii. Inconsistent or absent response  to external stimuli  iv. Decreased arousability  v. Seizure associated with loss of  consciousness  OR  Focal or multifocal neurologic findings,  including one or more of the following:  a. Focal cortical signs  b. Cranial nerve abnormality  c. Visual field defect  d. Presence of primitive reflexes  e. Motor weakness  f. Sensory abnormality  g. Altered deep tendon reflexes  h. Cerebellar dysfunction | Fever | Pleocytosis  (WBC > 5/mm3  if ≥ 2 months  of age or  WBC >15  cells/mm3 if  < 2 months  of age) | Abnormality consistent with encephalitis | Abnormality consistent with encephalitis | Diagnostic certainty:  Level 1: Demonstration of acute inflammation of CNS parenchyma by histopathology  Level 2: Mandatory neurologic finding and >2 additional criteria  Level 3: Mandatory neurologic finding and 1 additional criterion | ^3^ |
| Encephalitis  Alternative 3 | Acute signs of parenchymatous brain dysfunction, such as (at least **one** of the following):  1. Signs of focal neurological deficit and/or  2. Lowered consciousness and disorientation  3. Seizures | Fever > 38°C | Pleocytosis  (WBC > 4/mm3) | - | Abnormality consistent with encephalitis | **One** of “mandatory findings”  **and**  At least **two** of the other findings (clinical/CSF/EEG) | ^2^ |

* A positive VZV PCR in cerebrospinal fluid (and/or positive VZV IgG-index) at the time of diagnosis was mandatory to fulfil the criteria for meningitis and encephalitis

Supplementary Table 3. VZV-associated CNS diagnoses in register-based study. ICD-10 codes for inclusion of VZV-associated CNS infection cases between 2010 and 2019.

| VZV-associated CNS diagnosis | ICD-10 code | ICD-10 concomitant code required at the same visit | Cases* (n) |
| --- | --- | --- | --- |
| Encephalitis/myelitis caused by VZV | B011 | - | 182^a^ |
| Encephalitis/myelitis caused by herpes zoster | B020 | - | 416^a^ |
| Meningitis caused by VZV | B010 | - | 280^b^ |
| Meningitis caused by herpes zoster | B021 | - | 429^b^ |
| Cerebellitis caused by VZV | B018 | G948 | 0 |
| Cerebellitis caused by herpes zoster | B028 | G948 | 0 |
| Facial palsy/Bell’s paresis caused by VZV (RHS) | B018 | G510 | 25^c^ |
| Facial palsy/Bell’s paresis caused by herpes zoster (RHS) | B028 | G510 | 228^c^ |
| Vasculitis caused by VZV | B018 | I778 | 0 |
| Vasculitis caused by herpes zoster | B028 | I778 | 1 |

Abbreviations: CNS, central nervous system; ICD-10, International Classification of Diseases 10th Revision; RHS, Ramsay Hunt syndrome; VZV, varicella-zoster virus

* Cases with both a diagnosis of encephalitis and meningitis or RHS are only counted as cases of encephalitis

^a^ 27 cases received both ICD-10 codes (B011 and B020)

^b^ 40 cases received both ICD-10 codes (B010 and B021)

^c^ 1 case received both ICD-10 codes (B018 and B028)

Supplementary Table 4. Definitions of comorbidities. ICD-10 codes and time frames used to identify comorbidities and prescribed medications

| Variable | ICD-10 code | Time before index date (years) |
| --- | --- | --- |
| HIV | B20–24 | No limit |
| Solid cancer | C00–26, C30–41, C45–58, C60–80, C97 | 5 |
| Hematological cancer | C81–86, C88, C90–96 | 10 |
| Lymphoma | C81–86, C88 | 10 |
| Myeloma | C90 | 10 |
| Leukemia | C91–95 | 10 |
| Transplantation | Z940–944, Z948–949 | No limit |
| Primary immunodeficiency | D80–84 | 10 |
| Diabetes | E10–14 | 10 |
| Chronic obstructive pulmonary disease | J449 | 5 |
| Alcohol abuse | F10 | 5 |
| Depression | F32 | 5 |
| Stroke | I61, I63–64 | 10 |
| Bleeding | I61 | 10 |
| Ischemia | I63 | 10 |
| Unspecified | I64 | 10 |
| Ischemic heart disease | I200, I21–I22 | 5 |
| Congestive heart failure | I50 | 10 |
| Asthma | J45 | 5 |

Abbreviations: HIV, human immunodeficiency virus; ICD-10, International Classification of Diseases 10th Revision

In order to be included, one of the listed ICD-10 codes was required to appear at least once within the given time limit before the index date. Supplementary Table 5. Immunosuppressive drugs. ATC codes and time frames used to identify prescribed medications

| Variable | ATC codes | Time before index date | Comment |
| --- | --- | --- | --- |
| Immunosuppressive drug | Includes all below |  |  |
| Glucocorticoids | H02AB | 1 year–30 days before | Treatment less than 30 days before index date was excluded to avoid confounding with VZV treatment |
| High-dose* | H02AB | 1 year–30 days before |  |
| Low and unknown dose* | H02AB | 1 year–30 days before |  |
| Antineoplastic agents | L01 | 1 year |  |
| Monoclonal antibodies | L01F | 1 year |  |
| Rituximab | L01FA01 | 1 year |  |
| Cyclophosphamide | L01AA01 | 1 year |  |
| Immunosuppressants | L04A | 1 year |  |
| Azathioprine | L04AX01 | 1 year |  |
| TNF-α inhibitors | L04AB01–07 | 1 year |  |
| Leflunomide | L04AA13 | 1 year |  |
| Alemtuzumab | L04AA34 | 1 year |  |
| Ciclosporin | L04AD01 | 1 year |  |
| Fingolimod | L04AA27 | 1 year |  |
| Natalizumab | L04AA23 | 1 year |  |
| IL inhibitors | L04AC | 1 year |  |
| Ixekizumab | L04AC13 | 1 year |  |
| Methotrexate | L04AX03 L01BA01 | 1 year |  |
| JAK inhibitors | L04AA29 L04AA37 L04AA44 L04AA45 L01EJ01 D11AH08 | 1 year |  |

Abbreviations: ATC, Anatomical Therapeutic Chemical; IL, interleukin; TNF-α, tumor necrosis factor alpha; VZV, varicella-zoster virus

*High-dose was defined as average daily dose of > 10 mg prednisolone equivalents, regardless of age and weight, based on Liu et al.^4^ Average daily dose was calculated as total dose of last filled prescription before index date divided by days until first filled prescription after index date. When this could not be calculated, dosage was considered to be unknown.

Supplementary Table 6. Antiviral drugs. ATC codes and time frames used to identify prescribed medications

| Variable | ATC codes | Time before index date | Time after index date |
| --- | --- | --- | --- |
| Aciclovir | J05AB01 | 30 days | 30 days |
| Valaciclovir | J05AB11 | 30 days | 30 days |
| Famciclovir | J05AB09 | 30 days | 30 days |

Abbreviations: ATC, Anatomical Therapeutic Chemical

*Positive predictive values*

A total of 114 medical records were reviewed and diagnosed as encephalitis (n = 40), meningitis (n = 40), or RHS (n = 34). Positive predictive values (PPV) for meeting predefined diagnosis criteria were calculated (Supplementary Table 7). In addition, some cases not reaching those criteria were considered probable after review of records. PPV including these are presented separately along with rationale for inclusion. Taken together with probable cases, overall PPV for any VZV-associated CNS infection when diagnosed by either encephalitis, meningitis, or RHS was 89% (95% CI [82–94]).

# Supplementary Table 7. Positive predictive values for VZV-associated CNS infection ICD-10 codes

|  | Encephalitis  n (PPV [95% CI]) | Meningitis  n (PPV [95% CI]) | RHS  n (PPV [95% CI]) |
| --- | --- | --- | --- |
| Cases reviewed | 40 | 40 | 34 |
|  |  |  |  |
| Criteria fulfilled | 29 (73% [58–84]) | 23 (58% [42–72]) | 30 (88% [74–96]) |
| Including considered probable* | 31 (78% [63–88]) | 24 (60% [45–74]) | 30 (88% [74–96]) |
| Including any VZV-associated CNS infection* | 37 (93% [81–98]) | 34 (85% [72–94]) | 30 (88% [74–96]) |
|  |  |  |  |
| VZV infection (not CNS) | 1 (3% [0–11]) | 6 (15% [7–28]) | 0 (0) |
| Other diagnosis | 2 (5% [1–15]) | 0 (0) | 4 (12% [4–26]) |
| Abbreviations: CI, confidence interval; CNS, central nervous system; ICD-10, International Classification of Diseases 10th Revision; PPV, positive predictive value; VZV, varicella-zoster virus  * Including cases not fulfilling predefined criteria, but included as probable cases after review of medical records | | | |

*Encephalitis*

In total, 29 of 40 (PPV 73%) encephalitis cases met the predefined criteria (Supplementary Table 1). PPV was 77% when adding cases that did not fulfil criteria, but were included as probable encephalitis after review of medical records (Supplementary Tables 8 and 9). Another six cases presented with other VZV-associated CNS infections (four meningitis and two RHS). Of the 29 cases meeting any set of predefined criteria, 11 would have been excluded by at least one other set of criteria (Supplementary Table 9). When comparing the sets of criteria, those according to Venkatesan 2013 would have excluded the most cases (10 of 29; 34%), whereas Sejvar 2007 would only exclude 2 of 29 (7%).

Supplementary Table 8. Probable encephalitis. Description of cases of encephalitis that did not fulfil predefined criteria, but were considered probable encephalitis after review of medical records.

| Age | Sex | Case description |  |  |
| --- | --- | --- | --- | --- |
| 84 | F | Herpes zoster on the arm, PCR from blisters positive. Fever, disorientation > 24 hours, decreased consciousness. No lumbar puncture performed. | | |
| 91 | F | Zoster ophthalmicus, seizures, fever. No lumbar puncture performed. | | |

Abbreviations: CSF, cerebrospinal fluid; PCR, polymerase chain reaction; VZV, varicella-zoster virus

# Supplementary Table 9. Encephalitis criteria fulfilment for three sets of criteria

| ID | Sex | Age | Enc crit 1 (Venkatesan) | Enc crit 2  (Sejvar) | Enc crit 3  (Grahn) |
| --- | --- | --- | --- | --- | --- |
| 1 | M | 24 | 0 | 0 | 2 |
| 2 | M | 75 | 0 | 0 | 2 |
| 3 | F | 88 | 0 | 1 | 0 |
| 4 | F | 85 | 0 | 1 | 0 |
| 5 | M | 62 | 0 | 1 | 0 |
| 6 | M | 84 | 0 | 1 | 0 |
| 7 | F | 59 | 0 | 2 | 2 |
| 8 | F | 20 | 0 | 2 | 2 |
| 9 | M | 73 | 0 | 2 | 2 |
| 10 | F | 76 | 0 | 2 | 2 |
| 11 | M | 82 | 1 | 1 | 0 |
| 12 | M | 91 | 1 | 2 | 2 |
| 13 | M | 83 | 1 | 2 | 2 |
| 14 | M | 83 | 1 | 2 | 2 |
| 15 | F | 86 | 1 | 2 | 2 |
| 16 | M | 87 | 1 | 2 | 2 |
| 17 | M | 76 | 1 | 2 | 2 |
| 18 | F | 76 | 1 | 2 | 2 |
| 19 | F | 78 | 2 | 2 | 2 |
| 20 | F | 72 | 2 | 2 | 2 |
| 21 | F | 71 | 2 | 2 | 2 |
| 22 | M | 56 | 2 | 2 | 2 |
| 23 | F | 88 | 2 | 2 | 2 |
| 24 | F | 88 | 2 | 2 | 2 |
| 25 | M | 80 | 2 | 2 | 2 |
| 26 | F | 73 | 2 | 2 | 2 |
| 27 | F | 75 | 2 | 2 | 2 |
| 28 | F | 83 | 2 | 2 | 2 |
| 29 | M | 76 | 2 | 2 | 2 |

Abbreviations and codes: 0 = Criteria not fulfilled, 1 = Possible encephalitis, 2 = Probable encephalitis. Enc crit X = encephalitis definition according to 1: Venkatesan 2013,^1^ 2: Sejvar 2007,^3^ or 3: Grahn 2013.^2^

*Meningitis*

In total, 23 of 40 (PPV 58%) cases met the predefined criteria for meningitis (Supplementary Table 1). PPV was 60% when adding one case that did not fulfil the criteria, but was included as probable meningitis after review of medical records (Supplementary Tables 7 and 10). Ten cases had other VZV-associated CNS infections, of which five cases were encephalitis, four cases RHS, and one case had cranial nerve disease (sudden deafness) caused by VZV, yielding a PPV for any VZV-associated CNS infection of 85%.

Supplementary Table 10. Probable meningitis. Description of cases of meningitis that did not fulfil predefined criteria, but were considered probable after review of medical records.

| Age | Sex | Case description |  |  |
| --- | --- | --- | --- | --- |
| 41 | M | Reumatic disease, treatment with methothrexate and TNF-α inhibitor. Generalized zoster. Symptoms of meningitis including headache, nausea, light sensitivity, neck stiffness, and fever. No lumbar puncture performed. | | |

Abbreviations: CSF, cerebrospinal fluid; PCR, polymerase chain reaction; VZV, varicella-zoster virus

*Ramsay Hunt syndrome*

In total, 30 of 34 (PPV 88%) cases met the predefined criteria for RHS (Supplementary Table 1). No other cases were considered probable after review of medical records, and none were linked to another VZV-associated CNS infection. Only four cases diagnosed exclusively in the primary care sector could be found in the regional register between 2000 and 2021. Three out of these (75%) were considered correct (Supplementary Table 11).

Supplementary Table 11. Description of cases with RHS diagnosis made exclusively in the primary healthcare sector

|  | Cases n (%) |
| --- | --- |
| RHS cases | 4 (100) |
|  |  |
| Correct | 3 (75) |
| Considered Bell’s palsy after early lumbar puncture without pleocytosis. Later positive VZV PCR on blisters. | 2 (50) |
| Otolaryngologist had already diagnosed, but did not give correct ICD-code | 1 (25) |
|  |  |
| Incorrect | 1 (25) |
| Facial nerve palsy and ipsilateral pain in ear. No blisters. Recent exposure to varicella. No lumbar puncture. Did not fulfil criteria. | 1 (25) |

Abbreviations: PCR, polymerase chain reaction; VZV, varicella-zoster virus

**References**

1. Venkatesan A, Tunkel AR, Bloch KC, Lauring AS, Sejvar J, Bitnun A, et al. Case definitions, diagnostic algorithms, and priorities in encephalitis: consensus statement of the international encephalitis consortium. Clin Infect Dis. 2013 Oct;57(8):1114-28. PubMed PMID: 23861361. Pubmed Central PMCID: PMC3783060. Epub 2013/07/19. eng.

2. Grahn A, Hagberg L, Nilsson S, Blennow K, Zetterberg H, Studahl M. Cerebrospinal fluid biomarkers in patients with varicella-zoster virus CNS infections. J Neurol. 2013 Jul;260(7):1813-21. PubMed PMID: 23471614.

3. Sejvar JJ, Kohl KS, Bilynsky R, Blumberg D, Cvetkovich T, Galama J, et al. Encephalitis, myelitis, and acute disseminated encephalomyelitis (ADEM): case definitions and guidelines for collection, analysis, and presentation of immunization safety data. Vaccine. 2007 Aug 1;25(31):5771-92. PubMed PMID: 17570566. Epub 2007/06/16. eng.

4. Liu D, Ahmet A, Ward L, Krishnamoorthy P, Mandelcorn ED, Leigh R, et al. A practical guide to the monitoring and management of the complications of systemic corticosteroid therapy. Allergy Asthma Clin Immunol. 2013 Aug 15;9(1):30. PubMed PMID: 23947590. Pubmed Central PMCID: PMC3765115. Epub 20130815. eng.
